# Molecular epidemiology of *Mycobacterium tuberculosis* complex in Brussels, 2010–2013

**DOI:** 10.1371/journal.pone.0172554

**Published:** 2017-02-21

**Authors:** Christelle Vluggen, Karine Soetaert, Guido Groenen, Maryse Wanlin, Martine Spitaels, Wouter Arrazola de Oñate, Maryse Fauville-Dufaux, Claude Saegerman, Vanessa Mathys

**Affiliations:** 1 Bacterial Diseases Service, Operational Direction Communicable and Infectious Diseases, Scientific Institute of Public Health (WIV-ISP), Brussels, Belgium; 2 Belgian Lung and Tuberculosis Association, Brussels, Belgium; 3 Research Unit of Epidemiology and Risk Analysis applied to Veterinary Sciences (UREAR-ULg), Fundamental and Applied Research for Animal and Health (FARAH) Centre, Liège, Belgium; Institut Pasteur de Lille, FRANCE

## Abstract

The tuberculosis (TB) incidence rate in Brussels-Capital Region is 3-fold higher than in Belgium as a whole. Eight years after the realization of initial prospective population-based molecular epidemiology investigations in this Region, a similar study over the period 2010–2013 was conducted. TB strains isolated from 945 patients were submitted to genotyping by standardized 24-locus-MIRU-VNTR typing and spoligotyping. The phylogenetic analysis showed that the LAM (16.7%) and Haarlem (15.7%) branches are the two most prevalent TB lineages circulating in Brussels. Analysis of the MDR subgroup showed an association with Beijing strains (39.9%) and patients native of Eastern Europe (40.7%). Genotyping detected 113 clusters involving 321 patients, giving a recent transmission index of 22.9%. Molecular-guided epidemiological investigations and routine surveillance activities revealed family transmission or social contact for patients distributed over 34 clusters. Most of the patients were foreign-born (75.7%). However, cluster analysis revealed only limited trans-national transmission. Comparison with the previous study shows a stable epidemiological situation except for the mean age difference between Belgian-born and foreign-born patients which has disappeared. This study confirms that molecular epidemiology has become an important determinant for TB control programs. However, sufficient financial means need to be available to perform all required epidemiological investigations.

## Introduction

Tuberculosis (TB) remains a major public health concern, even in developed countries. According to the World Health Organisation (WHO), an estimated 10.4 million people developed TB in 2015 and 1.4 million died from the disease [1]. In Belgium, the incidence in 2015 was 8.8 cases per 100,000 inhabitants, explaining its status of low TB incidence country. However, TB incidence rate in Brussels-Capital Region (25.9/100,000) is almost 3-fold higher than for the whole country while more than two thirds of the patients are foreign-born [2]. Belgium is a migration crossroad. During the study period, immigration from Eastern and Central Europe was increasing [3].

Rapid detection, adequate therapy, and contact tracing are key elements to stop further transmission and control the disease. Genotyping of *Mycobacterium tuberculosis* complex (*M*. *tuberculosis*), the causative agent of TB, can help to control the spread of the disease. Indeed, it allows the detection of unsuspected transmission, the discovery of laboratory contamination and the differentiation between reactivation and relapse [4]. Moreover, genotyping may also be useful to guide contact investigations, identify risk populations and evaluate the effectiveness of TB control programs [4,5].

Since 2003, the Belgian TB National Reference Laboratory performs TB strain typing by using the two main available techniques, 24-locus-MIRU-VNTR and spoligotyping; both are polymerase chain reaction (PCR)-based methods. Spoligotyping detects the presence or absence of spacer sequences located between repetitive elements by PCR and reverse dot-blot analysis [6]. This technique is easy and cheap but, used alone, presents a lack of discriminatory power [7]. MIRU-VNTR is based on the analysis of 24 loci containing variable numbers of tandem repeats (VNTRs) of genetic elements called mycobacterial interspersed repetitive units (MIRUs) [8–10]. Genotyping using a combination of spoligotyping and MIRU-VNTR has been shown to be generally as effective to study transmission of TB in Western Europe populations as IS6110 RFLP, which was considered as the previous gold standard for molecular epidemiogy of TB [11–13].

From 2002 to 2005, an initial prospective population-based molecular epidemiology study in the Brussels-Capital Region has been realised [11,14]. Genetic lineages of the *M*. *tuberculosis* strains were determined based on the spoligotypes and patient clustering was detected through the analysis of 24-locus-MIRU-VNTR and spoligotyping patterns. Eight years later, a similar study over 48 months was conducted in order to evaluate the evolution of the epidemiological situation, the *M*. *tuberculosis* transmission rate in Brussels, the effectiveness of recently implemented measures for improving surveillance as well as treatment access and follow-up of patients.

## Materials and methods

### Study subjects and design

From 1 January 2010 to 31 December 2013, the five hospitals of the Brussels-Capital Region that perform mycobacterial cultures and the Scientific Institute of Public Health (WIV-ISP), Brussels, Belgium, sent all *M*. *tuberculosis* cultures to the Tuberculosis and Mycobacteria Reference Center at the WIV-ISP, where one isolate from each patient was genotyped. Species identification was realized by the participating laboratories/hospitals using different techniques including PCR amplification of the IS*6110* element, MGIT^TM^ TBc Identification test (Becton Dickinson, Sparks, MD), SD Bioline TB Ag MPT64 Rapid test (Standard Diagnostics, Yongin, Korea) and Xpert® MTB/RIF assay (Cepheid, Sunnyvale, CA, USA). Drug susceptibility testing was conducted by the participating laboratories using the MGIT^TM^ method (Bactec^TM^, BD).

### Molecular typing method and analysis of patterns

The *M*. *tuberculosis* strains were submitted to genotyping by two different methods performed as previously described: spoligotyping [6] and standardized 24-locus-MIRU-VNTR typing using a four-capillary-based ABI3130-Avant genetic analyser [15,16]. Genetic lineages of the strains were determined by comparison of the obtained spoligotypes with those recorded at the www.miru-vntrplus.org [17,18]. Spoligotyping and MIRU-VNTR patterns were analyzed using the Bionumerics package, version 7.1 (Applied Maths, St-Martin-Latem, Belgium). Dendrograms were generated using the Pearson correlation and the unweighted-pair group method with arithmetic average linkages. A strain cluster was defined as two or more patients infected by isolates having identical spoligotypes and 24-locus-MIRU-VNTR patterns. Assuming that one patient from each strain cluster corresponded to the index case at the origin of infection, the strain-clustering rate (or recent transmission index, RTI) was calculated with the following equation: RTI_*n* − 1_ = (*n*_*c*_ − *c*)/*n*, where *n*_*c*_ is the total number of strain-clustered cases, *c* is the number of strain clusters, and *n* is the total number of cases in the sample [11,19].

### Patients’ information and epidemiological investigation

Some clinical and laboratory information (smear microscopy, site of infection, drug susceptibility testing and ambulatory/hospitalized patient status) were obtained through a questionnaire completed by the participating hospitals. The other clinical and demographic information were collected from the Belgian Tuberculosis Register. Anonymous data were obtained on sex, date and country of birth, place of residence, diabetes status, HIV status, cancer, alcoholism, kidney disease, recent contact (<2 years) with a TB patient, previous TB diagnosis, and socioeconomic status. All data accessed in the context of the present study had not been collected for research purposes but as part of the routine data collection for epidemiological surveillance, as stated in the Public Register dated 25/04/1997 in accordance with article 18 of the law of 08/12/1992 of the Belgian Government regarding the protection of the privacy of the individual when dealing with personal data. The aforementioned registration in accordance with the Belgian Privacy Commission stipulates in its §9 that no written informed consent from the patients is required for the collection and analysis of epidemiological data and treatment success collected for a public health purpose. The analysis of the data was realized after anonymization. The study was approved by the ethic committee of the Erasme University Hospital, one of the participating institutions (reference P2010/027).

### Statistical analysis

The frequencies and proportions observed in the clustered and non-clustered TB cases and in the multidrug resistant (MDR) and non-MDR TB cases were compared using the Chi2 test. The relationship between TB status (cluster *versus* non-cluster) and different exploratory variables (Table 1) was assessed using the odds ratio (OR) that were determined by logistic regression. When complete separation (zero cells) occurred, the Firth logit regression was used allowing inference of ORs and 95% confidence interval [20]. Differences in age between clustered and non-clustered TB cases were assessed using a two-sample Wilcoxon rank-sum (Mann-Whitney) test. For all tests, a *P* values < 0.05 was considered significant.

**Table 1:** Characteristics of *Mycobacterium tuberculosis* complex strains isolated from tuberculosis patients living in Brussels and two subgroups; no-clustered and clustered strains, 2010–2013.

Category	All strains included in the study (n = 945)		Non-clustered strains (n = 624)		Clustered strains (n = 321)		Clustered versus non-clustered strains	
	Sample proportion	% of isolates	Sample proportion	% of isolates	Sample proportion	% of isolates	Odds ratio (95% CI)	*P*-value
Gender								
Male	608/945	64.34	383/624	61.38	225/321	70.09	1.47 (1.10–1.97)	0.008
Age group								
<15 years	38/945	4.02	14/624	2.24	24/321	7.48	3.53 (1.76–7.09)	0.001
15–29 years	276/945	29.21	177/624	28.37	99/321	30.84	1.15 (0.83–1.61)	0.40
30–44 years	346/945	36.61	233/624	37.34	113/321	35.20	Reference	-
45–59 years	162/945	17.14	111/624	17.79	51/321	15.89	0.95 (0.63–1.41)	0.79
>60 years	123/945	13.02	89/624	14.26	34/321	10.59	0.79 (0.50–1.24)	0.30
Site of infection								
Pulmonary	697/920	75.76	437/606	72.11	260/314	82.80	1.86 (1.32–2.62)	<0.001
Extrapulmonary	223/920	24.24	169/606	27.89	54/314	17.2	Reference	-
Smear microscopy positive	320/627	51.04	193/391	49.36	127/236	53.81	1.20(0.85;1.67)	0.28
Native country								
Belgium	207/850	24.35	107/557	19.21	100/293	33.90	2.18 (1.58–3.00)	<0.001
Other countries	643/850	75.65	450/557	80.79	193/293	65.87	Reference	-
North Africa	180/850	21.18						
Sub-Saharan Africa	192/850	22.59						
Asia–Oceania	114/850	13.41						
America	18/850	2.11						
East Europe	92/850	10.82						
Western and central Europe	47/850	5.53						
Genetic sublineage								
Beijing	37/910	4.07	25/589	4.24	12/321	3.74	0.59 (0.28–1.24)	0.17
ast Africa Indian	19/910	2.09	19/589	3.23	0/321	0.00	0.03 (0.002–0.52)	0.02
Delhi/CAS	51/910	5.60	35/589	5.94	16/321	4.98	0.51 (0.26–1.01)	0.05
LAM	152/910	16.70	83/589	14.09	69/321	21.50	Reference	-
Cameroon	19/910	2.09	12/589	2.04	7/321	2.18	0.72 (0.27–1.88)	0.50
TUR	2/910	0.22	2/589	0.34	0/321	0.00	0.24 (0.01–5.09)	0.36
Haarlem	143/910	15.71	85/589	14.43	58/321	18.07	0.82 (0.52–1.30)	0.40
NEW-1	1/910	0.11	1/589	0.17	0/321	0.00	0.40 (0.02–9.98)	0.58
S	30/910	3.30	15/589	2.55	15/321	4.67	1.20 (0.55–2.60)	0.64
X	19/910	2.09	17/589	2.89	2/321	0.62	0.18 (0.04–0.67)	0.01
URAL	56/910	6.15	23/589	3.91	33/321	10.28	1.71 (0.93–3.17)	0.09
Ghana	92/910	10.11	55/589	9.34	37/321	11.53	0.81 (0.48–1.37)	0.43
Uganda I	18/910	1.98	14/589	2.38	4/321	1.25	0.37 (0.12–1.13)	0.08
Uganda II	5/910	0.55	3/589	0.51	2/321	0.62	0.86 (0.16–4.49)	0.86
T or undefined	236/910	25.93	174/589	29.54	62/321	19.31	0.85 (0.51–1.41)	0.52
M. africanum	16/910	1.76	14/589	21.39	2/321	0.62	0.21 (0.05–0.82)	0.03
M. bovis	14/910	1.54	12/589	2.04	2/321	0.62	0.24 (0.06–0.97)	0.045
Drug resistance								
Isoniazid mono-resistance	45/922	4.88	24/606	3.96	21/316	6.65	1.72 (0.94–3.15)	0.08
Rifampicin mono-resistance	4/922	0.43	2/606	0.33	2/316	0.63	1.97 (0.28–14.05)	0.50
Multidrug resistance	30/922	3.25	21/606	3.47	9/316	2.85	0.84 (0.38–1.87)	0.67
Pan-susceptible	843/922	91.43	559/606	92.24	284/316	89.87	Reference	-
Previous history of TB	51/783	6.51	36/517	6.96	15/266 58/286	5.64	0,81(0,41;1,54)	0.48
Asylum-seekers or undocumented immigrants	214/861	24.85	156/575	27.13		20.28	0,68(0,48;0,97)	0.03
Prisoners	28/892	3.14	17/593	2.87	11/299	3.68	1.29 (0.60–2.80)	0.51
HIV positive	66/825	8.00	46/547	8.41	20/278	7.19	0.84 (0.49–1.46)	0.54
Immunosuppressive disease/treatment	36/836	4.31	26/556	4.68	10/280	3.57	0.75 (0.36–1.59)	0.46
Underprivileged	333/804	41.42	214/532	40.23	119/272	43.75	1.16 (0.86–1.55)	0.34
Alcohol abuser	49/821	5.97	24/548	4.38	25/273	9.16	2.20 (1.29–3.93)	0.008
Homeless	35/876	3.99	25/586 38/265	4.27	10/290	3.45	0.80 (0.38–1.69)	0.56
Contact < 2 years	95/408	23.28		14.34	57/143	39.86	3.96 (2.45–6.40)	<0.001
Denutrition	74/787	9.40	43/516	8.33	31/271	11.44	1.42 (0.87–2.31)	0.16
Renal disease	27/831	3.25	24/558	4.30	3/273	1.10	0.25 (0.07–0.83)	0.02
Cancer	13/831	1.56	7/553	1.27	6/278	2.16	1.72 (0.57–5.17)	0.33
Diabetes	20/835	2.39	15/556	2.70	5/279	1.79	0.66 (0.24–1.83)	0.42
Outcome 12 months								
Completed	389/670	58.06	257/433	59.35	132/237	55.70	0.93 (0.62–1.39)	0.70
Cured	140/670	20.90	90/433	20.79	50/237	21.10	Reference	-
Defaulted	116/670	17.31	72/433	16.63	44/237	18.67	1.10 (0.66–1.83)	0.71
Died tuberculosis	11/670	1.64	7/433	1.62	4/237	1.69	1.03 (0.29–3.69)	0.97
Died other morbidity	7/670	1.04	4/433	0.92	3/237	1.27	1.35 (0.29–6.27)	0.70
Died unknown	7/670	1.04	3/433	0.69	4/237	1.69	2.4 (0.52–11.15)	0.26

## Results

### Clinical isolates

From 1 January 2010 to 31 December 2013, the Belgian Lung and Tuberculosis Association (BELTA) registered 1342 TB cases in the Brussels-Capital Region, 1068 (79.6%) of them being culture-positive. The isolates from 954 (89.3%) of the culture-positive patients were sent to the National Reference Laboratory where one strain per patient was selected for inclusion in the study. Of these, nine cases were excluded because of laboratory cross-contamination (n = 7) and labeling/patient identification error (n = 2) as confirmed by genotyping and laboratory/clinical investigation (samples collected or processed the same day and/or absence of clinical TB symptoms). A total of 945 TB isolates were included in the study. Additional efforts to retrieve the 114 missing cultures were made unsuccessfully. As previously described, young patients (less than 15 years; n = 67) and patients with extrapulmonary TB (n = 139), known to give paucibacillary samples, were overrepresented among the culture-negative patients (data not shown) [2,14].

### Characteristics of TB patients

Information on native country was available for 850 of the 945 patients (Table 1): 24.4% (n = 207) of the Brussels TB patients were born in Belgium, the remaining 75.7% (n = 643) in 76 different countries, the majority (n = 372, 43.8%) being from Africa: 22.6% from Sub-Saharan Africa and 21.2% from North-Africa. The most frequently encountered native countries were Morocco (18.7%) and the Democratic Republic of Congo (6.6%). Among the TB patients living in Brussels, 24.9% were asylum-seekers or undocumented immigrants.

Most of the patients belonged to the active population age group with a mean age of 38.4 years (standard deviation (SD) = 18.0). No significant difference in age was observed between patients born in Belgium (mean age = 39.2 years, SD = 23.2) and foreign-borns (meanage = 38.0 years, SD = 16.0 years). Among Belgian-born patients the females (mean age 35.0 years) were younger than the males (mean age 41.5 years), but this difference was not significant. The overall sex ratio was 1.8 (608 males and 337 females). In very young children (< 2 years), boys (n = 18) were more often affected than girls (n = 7) (sex ratio: 2.6).

Information on the biological origin of the isolates was available for 920 patients. The vast majority were isolated from the respiratory tract (75.8%) followed by lymph nodes (15.4%), pleural fluid (3.4%) and other sites (see Table 1). A pulmonary infection site was significantly less frequent among foreign patients (72.8%) than among Belgian ones (82.0%) (p = 0.004) while lymphatic infection was significantly more frequent among the former (18.0% versus 9.8%, p = 0.006).

At the time of sampling, 66.0% of the patients were hospitalized and 51.0% of pulmonary cases were smear microscopy positive (see Table 1). For 6.5% of the patients (n = 51), a previous history of TB was registered. These relapses occurred on average 9.2 years (SD = 9.3) after the first TB occurrence (data available for 43 of the 51 patients).

Analysis of risk factors revealed that 6.0%, 3.1%, 41.4% of the patients were registered as alcohol abuser, prisoner and/or underprivileged, respectively. Moreover, HIV-positive patients accounted for 8.0% of the study population and 9.4% were notified as suffering from malnutrition. For 23.3% of the patients a recent contact with a TB patient was recorded.

At the end of the study period, treatment outcome at 12 months was available for 716 patients; 46 were still on treatment. Out of the 670 patients remaining, 529 (79.0%) had completed their treatment successfully. There are no significant differences between the clustered and non-clustered TB cases regarding these parameters.

### Molecular typing and clustering analysis

The 945 TB isolates included in this study were submitted to genotyping by standardized MIRU-VNTR typing and spoligotyping. Genotypes were interpretable for 910 strains (S1 File). The majority (n = 880) of the TB cases were infected by *M*. *tuberculosis* but 16 were infected by *M*. *africanum* and 14 by *M*. *bovis*. The distribution of the *M*. *tuberculosis* genetic families is presented in Table 1. The large majority of the strains belonged to the Euro-American lineage, with sublineages Latin-American-Mediterranean (LAM) (16.7%) and Haarlem (15.7%) being the most prevalent.

Of the 910 isolates, only 6 reproducibly displayed a double allele in a single MIRU-VNTR locus each (MIRU 27, VNTR 1955, VNTR 0424, VNTR 4052, VNTR 2163b, MIRU 10). For these isolates, the double allele was considered for possible overlaps of clusters identification. Two of them were involved in one cluster each and included in this study. The combined use of spoligotyping and MIRU-VNTR for the cluster analysis had no effect on the resolution power obtained by using MIRU-VNTR typing alone.

Cluster analysis, applied to the 910 genotypable isolates, allowed the identification of 113 clusters involving 321 (35.3%) of the TB patients. These results yield a strain-clustering rate of 22.9%. Cluster size ranged from 2 to 11 patients with the following distribution: cluster of 2 patients (n = 68), 3 (n = 25), 4 (n = 7), 5 (n = 6), 6 (n = 4), 7 (n = 1), 10 (n = 1) and 11 (n = 1). Similar to the global population, the sublineages LAM (21.5%) and Haarlem (18.1%) were the most prevalent among the clustered strains. Of the 589 patients whose strain did not belong to any cluster, 80.8% were foreign-born.

No significant difference in the percentage of smear positive samples was observed between patients involved in a cluster and patients infected by an isolate presenting a unique pattern. However, more pulmonary infections (82.3% versus 72.1%, p<0.01) and Belgian-born persons (33.9% versus 19.2%, p<0.01) were observed among patients involved in clusters. Moreover, more males (p = 0.008) and children (less than 15 years, p<0.001) were observed in this clustered population. Among the children, the majority (17/24) were under 3 years of age and had clear familial or contact links with the other members of their cluster. Being an alcohol abuser (p = 0.008) or having had a recent contact with a TB patient (p<0.001) were also a strain-clustering predictor.

Although 100 of the 321 patients involved in clusters were Belgian-born, epidemiological investigations could establish a foreign connection (e.g. foreign parents or wife/husband, frequent journeys abroad) for 33 among them. Globally, 10 clusters comprised exclusively Belgian-born patients (n = 27), 57 consisted of only foreign-born patients (n = 134) and 46 presented a mix of both (n = 160). In most of the mixed clusters (n = 41), the foreign-born patients were long-time residents in Belgium. Only 5 clusters presented a mix of Belgian-born patients and recently arrived asylum-seekers or unsettled (often undocumented) immigrants.

**Fig 1:**
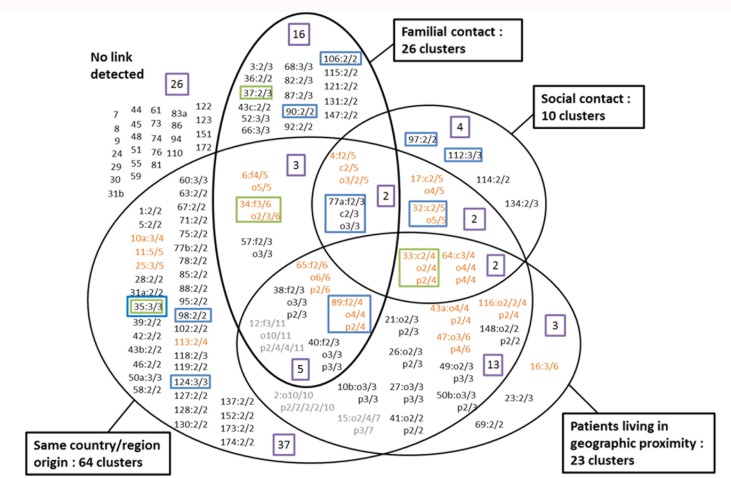
Repartition of the clusters according to the links identified between the patients. Clusters are represented by unique identification numbers. Next to the cluster number, the number of patients for who an epidemiological link was detected is indicated (i.e. 2/3: two of the three patients included in the cluster present this link). f: familial link; o: same geographic origin; p: geographic proximity. Clusters in orange contain more than 3 patients and cluster in grey more than 6. The purple boxes indicate the number of clusters belonging to each category (or combination of categories) of links. The blue boxes indicate clusters comprising exclusively Belgian-born patients. The green boxes indicate clusters with strains presenting the 776000000000171 spoligotype (S-family).

Analysis of information collected by the field staff of the two Belgian TB associations, VRGT and FARES, during molecular-guided epidemiological investigations, supplemented by data obtained during routine surveillance activities, revealed family transmission or social contact for 58 and 21 patients respectively, distributed over 34 clusters. Patients with undetermined links but living in geographic proximity and patients presenting only links of geographical origin were included in 23 (63 patients) and 64 (107 patients) clusters respectively. Distribution of the strain-clusters according to the epidemiologic links identified between the involved patients is presented in Fig 1. No obvious link was detected for 72 patients.

Isolates presenting the spoligotype 776000000000171, identified in 2003–2005 as responsible for a large outbreak (41 patients) in Brussels [11] were again detected in this study. Eighteen patients were infected, of whom 15 were included in 4 clusters. Among these 15 strain-clustered patients, 9 were Belgian-born and 9 had confirmed familial (n = 7) or social (n = 2) links.

One of the 4 clusters contained strains presenting also the same MIRU-VNTR profile as those analysed during the Brussels outbreak. Two clusters differed by variations in a single locus (VNTR 4052) and the fourth one presented a divergent MIRU-VNTR profile with variations in 5 loci (MIRU 40, VNTR 2163b, MIRU 16, VNTR 4052 and VNTR 2165). Concerning the three patients sharing this spoligotype but not involved in clusters, variations in the MIRU-VNTR profiles were observed, respectively, in a single locus (n = 1), four loci (n = 1) and 18 loci (n = 1).

### Drug susceptibility

Drug susceptibility results were available for 922 of the 945 isolates included in the study. The vast majority (91.4%) were susceptible to isoniazid and rifampicin, the two main antibiotics used for TB treatment. However, 30 strains (3.3%) showed resistance to these two drugs and were, therefore, identified as multi-drug resistant (MDR) strains. Among these, 4 were considered extensively drug resistant (XDR) presenting additional resistance to any fluoroquinolone and to at least one injectable second-line drug. Comparing the MDR subgroup of patients to those infected by a susceptible TB strain, the former present a significantly higher frequency of previous history of TB (65.4% against 5.6%; p<0.001). Pulmonary infections were also more frequent in the MDR subgroup of patients compared to those infected by a susceptible TB strain (89.7% against 75.3%) but this difference was not significant (p = 0.09). Out of the 30 MDR TB patients, 13 had not yet completed their treatment at the end of the study period. Among the 17 who did, 15 were cured, a cure rate of 88.2%. One patient died, and 1 was lost to follow-up.

The analysis of the genetic lineage among the MDR subgroup showed a strong presence of strains belonging to the Beijing family (39.9% versus 2.9% in the non-MDR patients; p = 0.049) while strains of the Haarlem family are less frequent in this subgroup (3.3% versus 16.2%; p = 0.57). Analysis of the native countries showed that MDR patients are mainly foreign-born (92.6%) with many (40.7%) originating from Eastern Europe, against only 9.8% among the non-MDR patients (p = 0.001). These two observations are linked as MDR patients infected by a Beijing strain (n = 12) are nearly all (10/12) natives from Eastern European countries: Georgia (n = 5), Latvia (n = 1), Poland (n = 1) and Russia (n = 3).

## Discussion

This report presents the conclusion of a population-based molecular epidemiology study conducted over the period 2010–2013 on 945 positive cultures TB patients living in Brussels-Capital Region. The proportion of the TB patients in Brussels that is foreign-born (75.7%) has hardly changed when compared to a similar epidemiological study in Brussels realized eight years earlier (2002–2005) which found a proportion of foreign-born TB patients of 78.8% [11].

The two studies also show an evolution of the difference in patients’ age distribution between Belgian-born and foreign-born patients. The previous study revealed a substantial difference in the mean age of Belgian-born (51 years) and foreign-born (32 years) patients while this difference seems to have been erased in the present study, with mean ages of 39.3 and 38.4 years, respectively [21]. This observation could be linked to the progressive disappearance of Belgian-born people infected at a young age who developed an active tuberculosis later (birth-cohort effect). Public Health campaigns promoting hygiene, the increase of the standard of living, social protection mechanisms, improved working conditions [22] and the introduction of antibiotics in the treatment during the post-war period are key elements explaining this reduction of infection. At the national level, a similar decrease of TB among persons over 65 years of age was reported and seems to be related to a cohort effect mainly observed in the Belgian population [2].

The study identified classic risk factors for TB transmission such as being underprivileged (41.4%), having a recent contact with a TB patient (23.2%) or being asylum-seekers or undocumented immigrants (24.9%). Having a previous history of TB was also confirmed to be a MDR TB predictor. It should be noted that the definition of some of the risk factors is not precise and the data should therefore be interpreted with caution.

The treatment success rate obtained in the study population in the Brussels-Capital Region (79.0%) is comparable to the result obtained in the rest of the country (77.3% average of the cohorts 2010–2013 in the Flemish and Walloon regions) (reference national TB registers 2011–2014).

The cure rate of 88.2% among 17 MDR TB patients in the study in Brussels-Capital Region is comparable to the 83.7% obtained in the MDR cohorts 2010–2013 in the other regions of the country (Flanders and Wallonia) [23].

The phylogenetic analysis of the isolates showed that the LAM (16.7%) and Haarlem (15.7%) branches are the two most prevalent TB lineages circulating in Brussels. However, analysis of the MDR subgroup showed an association with Beijing strains (39.9%) and patients native of East-Europe (40.7%), as also reported globally in Belgium [23,24] and in the European Union [25,26]. These observations were also mentioned during the previous epidemiological study conducted in Brussels, indicating a stable phylogenetic distribution of the strains. In 2003–2005, an outbreak including 41 patients infected by a strain belonging to the S genetic family and presenting the spoligotype 776000000000171 (octal code) was described [11]. In the present study, 18 additional strains presenting this pattern were identified, showing that the strain S-Brussels is still actively transmitted in the community about 8 years after its first detection and that MIRU-VNTR variants are emerging.

Genotyping of the TB strains allowed the detection of 113 clusters involving 321 patients, giving a recent transmission index of 22.9% compared to 19.8% reported previously (2002–2005) [14]. These data illustrate the stability of the TB context in the Brussels-Capital Region but also prove the necessity of remaining vigilant and maintaining the TB control efforts. As most of the TB patients in Brussels-Capital Region are foreign-born, it is likely that many among them were infected in their native country. Arguments in favour of this assumption are that proportion-wise, significantly more Belgian-born than foreign-born patients belonged to a cluster while, inversely, the latter were infected more often by an isolate with a unique genetic profile. The fact that genotyping revealed that the strain of 64.7% of the patients (n = 589) presented a unique genotype demonstrates the large genetic diversity of the TB strains isolated from patients living in Brussels.

Among the 113 clusters that were detected, 57 (50.4%) consisted of foreign-born patients only while 46 (40.7%) constituted a mix of Belgian-born and foreign-born patients. In the earlier study, these percentages were 58.5% and 32.1% respectively, suggesting a shift from foreign-born only to mixed clusters. However, in both studies, the foreign-borns in most of the mixed clusters were settled immigrants who were long-time residents in the country. Only 3 out of 17 mixed clusters in the previous study and 5 out of 46 in the present study presented a mix of Belgian-born patients and recently arrived asylum-seekers or unsettled immigrants, revealing limited cross-national TB transmission [27].

Additional investigations of the 113 clusters detected during the present study showed evident links, such as familial/social contact, same geographic origin or geographic proximity of the places of residence, within 87 clusters. By using only conventional epidemiological surveillance (based on routinely collected information such as recent contact with a TB patient or familial/social links) and not genotyping, a clear link would only have been established for 19.3% of the strain-clustered patients. This analysis confirms that molecular epidemiology of *M*. *tuberculosis* has become an important determinant for TB control programs. Moreover, with many laboratories currently implementing new technologies such as whole genome sequencing, a better discriminatory power among clusters will soon be possible [26].

In addition to detecting laboratory cross contamination and clerical errors, molecular surveillance has led to the identification of unsuspected transmission chains and, accordingly, the subsequent development of targeted interventions [28]. In this way, the detection of patient clusters is helping the Public Health Organisations to respond to TB outbreaks more effectively and improve TB management and control in the country.

To enjoy the benefit of molecular surveillance in practice, a close collaboration between laboratories and public healthcare workers is needed to quickly perform genotyping after TB culture positivity and implement control measures as soon as the result is available. However, during the present study it came to the fore that the main obstacle to a TB control strategy based on molecular surveillance in Belgium are the budgetary restrictions that do not allow to carry out extensive field investigations and contact tracing for each of the clusters identified. Another consequence of the current limited financial means is the impossibility to genotype all the TB strains isolated in Belgium although a country-wide monitoring has been proven to be more efficient [28].

Given the high cost required to treat a TB patient, as evidenced by different studies [23,29], a financial investment in molecular surveillance and the epidemiological investigations it entails would be economically favourable.

## Conclusion

The TB epidemiological situation in the Brussels-Capital Region (Belgium) has remained stable between the periods 2002–2005 and 2010–2013, with the exception of the mean age difference between Belgian-born and foreign-born patients which probably has disappeared as a result of a cohort effect.

Cluster analysis has revealed that only limited trans-national transmission is taking place, but it is clear that one country on its own will not be able to eliminate TB within its territory. A global approach to TB control is required.

Molecular surveillance has a clear added value as it allows to identify clustering, laboratory contamination, clerical errors and unsuspected transmission chains. But sufficient financial means need to be available to perform all required epidemiological investigations to establish linkages and to genotype all the TB strains isolated in the country.

## Supporting information

S1 File MIRU-VNTR typing and spoligotyping results. (XLS)

## References

[1] World Health Organisation. Global Tuberculosis Report. WHO/HTM/TB/2016.13. 2015.

[2] FARES. Registre de la tuberculose 2015. 2016.

[3] Martiniello M, Mazzocchetti J, Rea A. Editorial: Les nouveaux enjeux des migrations en Belgique. Revue européenne des migrations internationales 2/2013 2016;29:7–14.

[4] Barnes PF, Cave MD. Molecular epidemiology of tuberculosis. N Engl J Med 2003 9 18;349(12):1149–56. 10.1056/NEJMra021964 13679530

[5] Moss AR, Hahn JA, Tulsky JP, Daley CL, Small PM, Hopewell PC. Tuberculosis in the homeless. A prospective study. Am J Respir Crit Care Med 2000 8;162(2 Pt 1):460–4. 10934071 10.1164/ajrccm.162.2.9910055

[6] Kamerbeek J, Schouls L, Kolk A, van Agterveld M, van Soolingen D, Kuijper S, et al Simultaneous detection and strain differentiation of Mycobacterium tuberculosis for diagnosis and epidemiology. J Clin Microbiol 1997 4;35(4):907–14. 9157152 10.1128/jcm.35.4.907-914.1997 PMC229700

[7] van Soolingen D. Molecular epidemiology of tuberculosis and other mycobacterial infections: main methodologies and achievements. J Intern Med 2001 1;249(1):1–26. 11168781 10.1046/j.1365-2796.2001.00772.x

[8] Frothingham R, Meeker-O'Connell WA. Genetic diversity in the Mycobacterium tuberculosis complex based on variable numbers of tandem DNA repeats. Microbiology 1998 5;144 (Pt 5):1189–96. 9611793 10.1099/00221287-144-5-1189

[9] Supply P, Magdalena J, Himpens S, Locht C. Identification of novel intergenic repetitive units in a mycobacterial two-component system operon. Mol Microbiol 1997 12;26(5):991–1003. 9426136 10.1046/j.1365-2958.1997.6361999.x

[10] Supply P, Mazars E, Lesjean S, Vincent V, Gicquel B, Locht C. Variable human minisatellite-like regions in the Mycobacterium tuberculosis genome. Mol Microbiol 2000 5;36(3):762–71. 10844663 10.1046/j.1365-2958.2000.01905.x

[11] Allix-Beguec C, Supply P, Wanlin M, Bifani P, Fauville-Dufaux M. Standardised PCR-based molecular epidemiology of tuberculosis. Eur Respir J 2008 5;31(5):1077–84. 10.1183/09031936.00053307 18094006

[12] Cardoso OM, Gomes HM, Willery E, Possuelo L, Batista Lima KV, Allix-Beguec C, et al The forest behind the tree: phylogenetic exploration of a dominant Mycobacterium tuberculosis strain lineage from a high tuberculosis burden country. PLoS One 2011;6(3):e18256 10.1371/journal.pone.0018256 21464915 PMC3064675

[13] de Beer JL, van Ingen J, de Vries G, Erkens C, Sebek M, Mulder A, et al Comparative study of IS6110 restriction fragment length polymorphism and variable-number tandem-repeat typing of Mycobacterium tuberculosis isolates in the Netherlands, based on a 5-year nationwide survey. J Clin Microbiol 2013 4;51(4):1193–8. 10.1128/JCM.03061-12 23363841 PMC3666783

[14] Allix-Beguec C, Fauville-Dufaux M, Supply P. Three-year population-based evaluation of standardized mycobacterial interspersed repetitive-unit-variable-number tandem-repeat typing of Mycobacterium tuberculosis. J Clin Microbiol 2008 4;46(4):1398–406. 10.1128/JCM.02089-07 18234864 PMC2292969

[15] Allix C, Supply P, Fauville-Dufaux M. Utility of fast mycobacterial interspersed repetitive unit-variable number tandem repeat genotyping in clinical mycobacteriological analysis. Clin Infect Dis 2004 9 15;39(6):783–9. 10.1086/423383 15472808

[16] Supply P, Allix C, Lesjean S, Cardoso-Oelemann M, Rusch-Gerdes S, Willery E, et al Proposal for standardization of optimized mycobacterial interspersed repetitive unit-variable-number tandem repeat typing of Mycobacterium tuberculosis. J Clin Microbiol 2006 12;44(12):4498–510. 10.1128/JCM.01392-06 17005759 PMC1698431

[17] Weniger T, Krawczyk J, Supply P, Niemann S, Harmsen D. MIRU-VNTRplus: a web tool for polyphasic genotyping of Mycobacterium tuberculosis complex bacteria. Nucleic Acids Res 2010 7;38(Web Server issue):W326–W331. 10.1093/nar/gkq351 20457747 PMC2896200

[18] Allix-Beguec C, Harmsen D, Weniger T, Supply P, Niemann S. Evaluation and strategy for use of MIRU-VNTRplus, a multifunctional database for online analysis of genotyping data and phylogenetic identification of Mycobacterium tuberculosis complex isolates. J Clin Microbiol 2008 8;46(8):2692–9. 10.1128/JCM.00540-08 18550737 PMC2519508

[19] Small PM, McClenny NB, Singh SP, Schoolnik GK, Tompkins LS, Mickelsen PA. Molecular strain typing of Mycobacterium tuberculosis to confirm cross-contamination in the mycobacteriology laboratory and modification of procedures to minimize occurrence of false-positive cultures. J Clin Microbiol 1993 7;31(7):1677–82. 8102372 10.1128/jcm.31.7.1677-1682.1993 PMC265613

[20] Heinze G, Schemper M. A solution to the problem of separation in logistic regression. Stat Med 2002 8 30;21(16):2409–19. 10.1002/sim.1047 12210625

[21] Allix-Beguec C. Marqueurs génétiques du complexe Mycobacterium tuberculosis: études phylogénétiques et épidémiologiques de la tuberculose 2007.

[22] de Colombani P, Hovhannesyan A. Social determinants and risk factors for tuberculosis in national surveillance systems in Europe. Public Health Action 2015 9 21;5(3):194–201. 10.5588/pha.15.0026 26399291 PMC4576762

[23] Groenen G. BELTA-TBnet activity reports 2012–2015. Belgian Lung and Tuberculosis Association. Eendrachtstraat 56, 1050 Brussels, Belgium. 2015.

[24] Stoffels K, Allix-Beguec C, Groenen G, Wanlin M, Berkvens D, Mathys V, et al From multidrug- to extensively drug-resistant tuberculosis: upward trends as seen from a 15-year nationwide study. PLoS One 2013;8(5):e63128 10.1371/journal.pone.0063128 23671662 PMC3650045

[25] de Beer JL, Kodmon C, van der Werf MJ, van Ingen J, van Soolingen D. Molecular surveillance of multi- and extensively drug-resistant tuberculosis transmission in the European Union from 2003 to 2011. Euro Surveill 2014;19(11). 10.2807/1560-7917.es2014.19.11.20742 24679719

[26] Borgdorff MW, van Soolingen D. The re-emergence of tuberculosis: what have we learnt from molecular epidemiology? Clin Microbiol Infect 2013 10;19(10):889–901. 10.1111/1469-0691.12253 23731470

[27] Sandgren A, Schepisi MS, Sotgiu G, Huitric E, Migliori GB, Manissero D, et al Tuberculosis transmission between foreign- and native-born populations in the EU/EEA: a systematic review. Eur Respir J 2014 4;43(4):1159–71. 10.1183/09031936.00117213 24114966 PMC3971120

[28] Lambregts-van Weezenbeek CS, Sebek MM, van Gerven PJ, de Vries G, Verver S, Kalisvaart NA, et al Tuberculosis contact investigation and DNA fingerprint surveillance in The Netherlands: 6 years' experience with nation-wide cluster feedback and cluster monitoring. Int J Tuberc Lung Dis 2003 12;7(12 Suppl 3):S463–S470. 14677839

[29] Diel R, Vandeputte J, de Vries G, Stillo J, Wanlin M, Nienhaus A. Costs of tuberculosis disease in the European Union: a systematic analysis and cost calculation. Eur Respir J 2014 2;43(2):554–65. 10.1183/09031936.00079413 23949960

